# Identification of SFPQ as novel interacting partner of HIV-1 Integrase and its functional characterization

**DOI:** 10.1186/1471-2334-12-S1-P80

**Published:** 2012-05-04

**Authors:** Souvik Sur, Nirpendra Singh, Navrinder Kaur, Atul Ranjan, Braham Parkash, Ramesh Chandra, Vibha Tandon

**Affiliations:** 1 Department of Chemistry, University of Delhi, India; 2 Dr. B. R. Ambedkar Center for Biomedical Research, University of Delhi, India

## Background

HIV-1 requires the support of its appropriate host for the survival and propagation like any other parasite. These host cell factors have been reported to be involved in different stages of viral life cycle. The nuclear import and infection maintenance of HIV-1 Integrase (IN) in human cells is dependent upon the integration efficiency of the proviral DNA and stability of viral RNA. In our study we identified a new host cell interacting factor for HIV-1 IN, SFPQ-a RNA splicing protein, by cross-linking, pull down and mass spectrometry.

## Methods

This study involved gene cloning, protein expression, purification, cell culture, transfection and selection of stable cell lines for the HIV-1 IN GFP fusion protein and in-vitro IN activity assays study.

## Results

We identified a new host cell interacting factor for HIV-1 IN-SFPQ, an RNA splicing protein that interacts with HIV-1, shows 5 fold binding (K_d_ 0.05 µM) and modulates its disintegration activity.

## Conclusions

This study presents SFPQ as a novel cellular factor which has functional interaction with HIV-1 IN. SFPQ, revealed that this protein is not only recruited to the sites where viral genome integration takes place but also increases the disintegration activity and 3’ end processing activities of HIV-1 IN.

